# Impact of extended source‐to‐surface distances and respiratory motion on the precision of electron beam therapy

**DOI:** 10.1002/acm2.70301

**Published:** 2025-10-10

**Authors:** Takaaki Ito, Hiroyuki Kosaka, Yuya Yanagi, Yusuke Sakai, Hajime Monzen

**Affiliations:** ^1^ Department of Medical Physics Graduate School of Medical Sciences Kindai University Osakasayama‐shi Osaka Japan; ^2^ Department of Radiological Technology Kobe City Nishi‐Kobe Medical Center Kobe Hyogo Japan; ^3^ Present address: Department of Radiology Shiga University of Medical Science Hospital Otsu Shiga Japan

**Keywords:** electron beam therapy, extended source‐to‐surface distance, respiratory motion

## Abstract

**Purpose:**

In electron beam therapy targeting superficial thoracic and abdominal lesions, the source‐to‐surface distance (SSD) varies due to patient respiration. This study aimed to investigate the percentage depth dose (PDD) and off‐center ratio (OCR) of 6, 9, and 12 MeV electron beams at extended SSDs; and to assess the variations in dose caused by respiratory motion.

**Methods:**

PDDs and OCRs for 6, 9, and 12 MeV electron beams were measured using a Blue Phantom 2 with 5 cm circular aperture and 10 × 10 cm^2^ fields at SSDs ranging from 98 to 105 cm. The dose differences at the maximum depth (*d*_max_) were calculated relative to an SSD of 100 cm. We simulated patient respiratory motion of 0–4 cm during irradiation with 5 cm circular aperture and 10 × 10 cm^2^ fields by placing a water‐equivalent phantom with a parallel‐plate ionization chamber on a QUASAR Platform. The gantry angle was set to 270°, and the SSD was set to 100 cm. The dose differences at *d*_max_ were calculated relative to a motion amplitude of 0 cm.

**Results:**

Extending the SSD by 3 cm reduced the dose by > 5% for all energies. For a 6 MeV electron beam with a 5 cm circular aperture, extending the SSD to 105 cm resulted in a dose reduction of 13.42% and expansions of the field size by 2.0 mm and the penumbra by 4.2 mm. A respiratory motion amplitude of 3 cm resulted in dose variations of > 3% for all energies and field sizes. The largest dose difference of 5.36% was observed for a 6 MeV electron beam with a 5 cm circular aperture.

**Conclusions:**

The results demonstrate that extending the SSD by 3 cm reduced the dose by >5% for all energies, providing a useful quantitative benchmark for quality assurance in superficial electron therapy. Respiratory motion management may be warranted for electron beam therapy when respiratory‐induced SSD variations exceed 3 cm.

## 1 INTRODUCTION

Electron beams are commonly used to treat superficial tumors, such as keloids, because the dose decreases sharply beyond the tumor.^1^ In electron beam therapy for keloids, a low electron energy is utilized to increase the surface dose, and it is generally performed at a source‐to‐surface distance (SSD) of 100 cm.^1^, ^2^

However, in treating the lateral neck region, a patient's shoulder may obstruct the positioning of the electron applicator. When the SSD extends beyond 100 cm, it is necessary to evaluate changes in the output and dose distribution characteristics of the electron beam to reduce treatment uncertainties.^3^, ^4^, ^5^, ^6^, ^7^, ^8^, ^9^ It has been noted before that corrections to the dose rate at extended SSDs do not follow the inverse‐square law if the nominal value of the SSD (usually 100 cm) is used in refs. 3, 5, 7, 9, 10. O'Shea et al. reported that extending the SSD from 100 to 105 cm decreases the dose at the maximum depth (*d*_max_) of a 10 × 10 cm^2^ irradiation field by ≈ 10% and expands the penumbra of the field.^5^ Additionally, these effects are reported to be more pronounced for irradiation fields with smaller areas and lower energies.^3^, ^5^, ^7^

In previous studies, the SSD was assumed to be fixed during irradiation.^3^, ^4^, ^5^, ^6^, ^7^, ^8^, ^9^ Although, the SSD during electron beam irradiation of thoracic and abdominal regions varies due to diaphragmatic movement and chest wall expansion associated with patient respiration, the effect of this respiration‐induced SSD extension on the accuracy of irradiation has not been reported.

The aims of this study were to investigate the percentage depth dose (PDD) and off‐center ratio (OCR) at extended SSDs and to determine the effects of respiratory motion on the precision of electron beam therapy.

## 2 METHODS

### 2.1 Equipment

A TrueBeam radiotherapy system (Varian Medical Systems, Palo Alto, CA, USA) was used to produce electron beams with energies 6, 9, and 12 MeV at a dose rate of 1000 monitor units (MU)/min. The values of the *d*_max_ were 1.3, 2.1, and 2.8 cm for a 10 × 10 cm^2^ field at 6, 9, and 12 MeV, respectively. The PDD and OCR were obtained using the Blue Phantom 2 advanced three‐dimensional water phantom system (IBA Dosimetry GmbH, Schwarzenbruck, Germany). PPC05 and PPC40 parallel‐plate ionization chambers (IBA Dosimetry GmbH, Schwarzenbruck, Germany)^11^ and an electron field diode (IBA Dosimetry GmbH, Schwarzenbruck, Germany) were used as detectors. A RAMTEC Duo electrometer (TOYO MEDIC, Tokyo, Japan) was also used. We simulated patient respiratory motion during irradiation by placing on the QUASAR Heavy Duty Programmable Respiratory Motion Platform (QUASAR platform, Modus QA, London, Canada) either a water‐equivalent phantom (Tough Water Phantom; Kyoto Kagaku Co., Ltd, Kyoto, Japan) with a PPC40 detector inserted or an IC Profiler (ICP) two‐dimensional ionization chamber (Sun Nuclear Corporation, Melbourne, FL, USA). We selected the electron energies (6, 9, and 12 MeV) and field sizes (a 5 cm circular aperture and a 10 × 10 cm^2^ field) based on typical clinical parameters for keloid treatment, where lower energies and smaller fields are commonly employed to maximize surface dose while minimizing deeper tissue exposure. These conditions were strategically chosen because they represent scenarios where electron beam characteristics, influenced by increased scattering effects, deviate most significantly from photon beam behavior.

### 2.2 Percentage depth dose and off‐center ratio at extended SSDs

**FIGURE 1:**
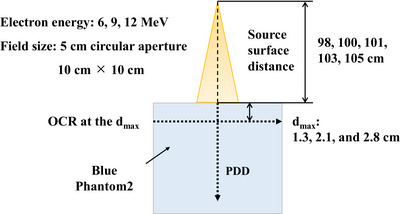
Schematic of the experimental geometries used to measure the percentage depth dose (PDD) and off‐center ratio (OCR) at each source‐to‐surface distance. *d*_max_, maximum depth.

The PDDs for 6, 9, and 12 MeV electron beams, as well as the OCRs at the *d*_max_ for these energies, were measured using the Blue Phantom 2 water phantom in 5 cm circular aperture and 10 × 10 cm^2^ fields at SSDs of 98, 100, 101, 103, and 105 cm. The values of the *d*_max_ were 1.3, 2.1, and 2.8 cm for a 10 × 10 cm^2^ field at 6, 9, and 12 MeV, respectively. We used the PPC05 detector to measure the PDD and the electron field diode to measure the OCR. The PDD and OCR were measured at each SSD and normalized relative to their respective values at *d*_max_ for an SSD of 100 cm (Figure 1). All measurements and subsequent conversions to radiation dose were performed following the Institute of Physics and Engineering in Medicine Code of Practice.^12^ The PDD and OCR values were compared for each energy, field size, and SSD. At the *d*_max_ for each energy, the differences in the PDD point dose at this depth for each SSD is compared to the value for the SSD of 100 cm value using the following calculation:

(1) $$\mathrm{Dose}\;\mathrm{Difference}\left(\%\right)=\frac{\mathrm{PD}D_{x}-\mathrm{PD}D_{100}}{\mathrm{PD}D_{100}}\times 100,$$

where PDD*_x_* and PDD_100_ correspond to the PDDs measured at SSDs of *x* and 100 cm, respectively. Additionally, to analyze OCRs, the field size and penumbra at the *d*_max_ were compared using their respective values at SSD = 100 cm as a reference. The penumbra was defined as the width of the off‐center distance between the 80% and 20% dose levels in each profile.

### 2.3 Effect of respiratory motion‐induced changes in SSD on irradiation accuracy

**FIGURE 2:**
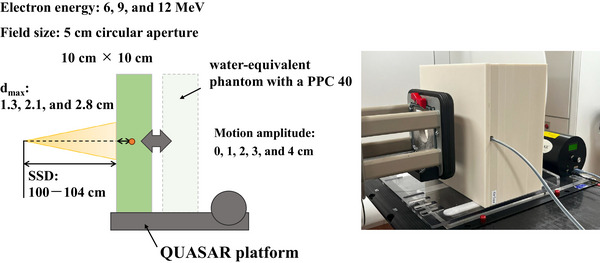
Schematic of the experimental setup for measurements of absorbed dose at the maximum depth (*d*_max_), using a PPC40 detector inserted in a water‐equivalent phantom placed on the QUASAR heavy duty respiratory motion platform.

**FIGURE 3:**
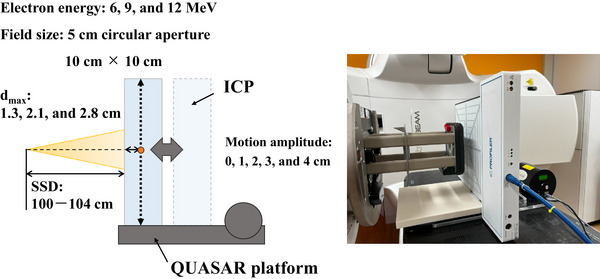
Schematic of the experimental setup for measurements of dose profiles using a two‐dimensional ionization chamber (IC Profiler) on the QUASAR heavy duty respiratory motion platform.

To measure the absorbed dose at the *d*_max_, a water‐equivalent phantom containing a PPC40 detector was placed on the QUASAR platform (Figure 2). The gantry angle was set to 270°, and the SSD was set to 100 cm. To simulate the respiratory motion of a typical adult patient, the respiratory cycle was set to 20 cycles/min, and the motion amplitude was set to 0, 1, 2, 3, and 4 cm.^13^ The minimum SSD in all cases was 100 cm. For each electron beam energy, a total dose of 20 Gy delivered in four fractions was simulated using 500 MU.^1^ Measurements were made at the *d*_max_ values for each energy in 5 cm circular aperture and 10 × 10 cm^2^ fields. The ICP was placed on the QUASAR platform (Figure 3) and used to characterize dose profiles under the same conditions used to measure the absorbed dose at the *d*_max_.

The absorbed dose at the *d*_max_ and dose profiles were measured for each energy, field size (5 cm circular aperture and 10 × 10 cm^2^), and amplitude of the QUASAR platform motion (0 to 4 cm). At the *d*_max_ value for each energy and field size, the difference in the absorbed doses between amplitudes of *x* cm and 0 cm (no motion) were calculated as:

(2) $$\mathrm{Dose}\;\mathrm{Difference}\left(\%\right)=\frac{\mathrm{dos}e_{x}-\mathrm{dos}e_{0}}{\mathrm{dos}e_{0}}\times 100,$$

where dose*_x_* and dose_0_ correspond to the relative dose measured at motion amplitudes of *x* and 0 cm, respectively. Additionally, to compare dose profiles, the field size and penumbra at the *d*_max_ were compared using their respective values in the absence of motion as a reference.

### 2.4 Statistical analysis

All data are presented as mean ± standard deviation. Pearson's correlation (*r*) was considered weak for *r* < 0.4, moderate for 0.4 ≤ *r* ≤ 0.7, and strong for *r* > 0.7.

## 3 RESULTS

### 3.1 PDD and OCR at extended SSDs

**FIGURE 4:**
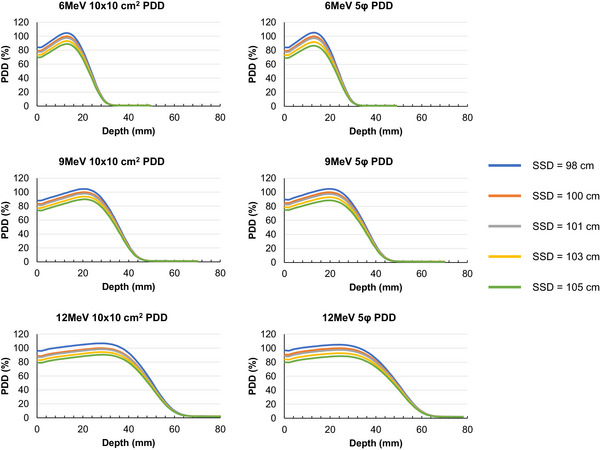
Comparison of the percentage depth dose (PDD) profiles for each source‐to‐surface distance (SSD) and electron energy with 5 cm circular aperture (5*φ*) and 10 × 10 cm^2^ field sizes.

The dose at the *d*_max_ decreased as a function of increasing SSD, with lower energies and smaller field sizes exhibiting greater decreases (Figure 4 and Table 1). When the SSD was increased by 3 cm from the 100 cm standard, the dose at the *d*_max_ was reduced by > 5% for all energies and field sizes. There was a correlation between the dose difference at the *d*_max_ due to extended SSDs and the extended distance of SSD for all energies and field sizes (*r* > 0.99).

**TABLE 1:** Comparison of the dose differences at the maximum depth (*d*_max_), calculated for each electron energy, field size, and source‐to‐surface distance (SSD).

Energy	Field size (cm^2^)	The dose differences at the *d*_max_			
		SSD 98 cm	SSD 101 cm	SSD 103 cm	SSD 105 cm
6 MeV	5 cm circular aperture	5.29%	−2.74%	−8.03%	−13.42%
9 MeV		4.83%	−2.47%	−7.00%	−11.54%
12 MeV		5.00%	−2.50%	−7.19%	−11.39%
6 MeV	10 × 10	4.70%	−2.45%	−6.85%	−11.06%
9 MeV		4.64%	−1.97%	−6.22%	−10.17%
12 MeV		6.85%	−1.69%	−5.66%	−9.43%

*Note*: Equation (1) was used to express the dose differences relative to the corresponding doses at an SSD of 100 cm.

The increase in field size and penumbra at the *d*_max_ for larger SSD is presented in Table 2. For electron energies of 6, 9, and 12 MeV with 5 cm circular aperture, extension of the SSD from 100 to 103 cm resulted in expansions of the field size by 1.2, 1.4, and 1.5 mm, respectively; for a field size of 10 × 10 cm^2^, the expansions were 3.4, 3.4, and 3.3 mm, respectively. Additionally, for 6, 9, and 12 MeV beams with 5 cm circular aperture, extending the SSD from 100 to 103 cm resulted in penumbra expansions of 2.6, 1.6, and 1.0 mm, respectively; for a field size of 10 × 10 cm^2^, the expansions were 2.6, 1.5, and 0.9 mm, respectively.

**TABLE 2:** Comparison of the differences in the irradiation field and penumbra sizes at the maximum depth (*d*_max_), calculated for different electron beam energies and field sizes, relative to an SSD of 100 cm.

		The differences of the irradiation field size on X‐axis (mm)				The differences of the penumbra on X‐axis (mm)			
Energy	Field size (cm^2^)	SSD 98 cm	SSD 101 cm	SSD 103 cm	SSD 105 cm	SSD 98 cm	SSD 101 cm	SSD 103 cm	SSD 105 cm
6 MeV	5 cm circular aperture	−0.8	0.4	1.2	2	−1.7	0.8	2.6	4.2
9 MeV		−0.9	0.4	1.4	2.2	−1	0.3	1.6	2.5
12 MeV		−1	0.5	1.5	2.4	−0.6	0.3	1	1.5
6 MeV	10 × 10	−2.3	1.1	3.4	5.7	−1.5	0.9	2.6	4.4
9 MeV		−2.1	1.3	3.4	5.6	−0.9	0.6	1.5	2.5
12 MeV		−2.2	1.1	3.3	5.6	−0.6	0.4	0.9	1.4

### 3.2 Effects of respiratory motion‐induced changes in SSD on irradiation accuracy

The dose differences between motion amplitudes of 0 cm (no motion) and 3 cm exceeded 3% for all energies and field sizes (Table 3). The largest dose difference (relative to no motion) was observed at 6 MeV with a 5 cm circular aperture. In contrast, for the 10 × 10 cm^2^ field size, the dose differences varied only slightly as a function of electron energy. There was a strong correlation between the dose difference and the amplitude of motion (*r* = 1.0).

**TABLE 3:** Comparison of differences in absorbed doses at the maximum depth (*d*_max_), calculated for different electron beam energies and field sizes, as a function of the amplitude of motion of the QUASAR platform.

		The dose differences at the *d*_max_			
Energy	Field size (cm^2^)	Motion amplitude 1 cm	Motion amplitude 2 cm	Motion amplitude 3 cm	Motion amplitude 4 cm
6 MeV	5 cm circular aperture	−1.36%	−2.64%	−4.05%	−5.36%
9 MeV		−1.17%	−2.32%	−3.38%	−4.51%
12 MeV		−1.12%	−2.15%	−3.23%	−4.24%
6 MeV	10 × 10	−1.05%	−2.10%	−3.18%	−4.20%
9 MeV		−1.04%	−2.07%	−3.15%	−4.10%
12 MeV		−1.03%	−2.09%	−3.13%	−4.08%

*Note*: Equation (2) was used to express the dose differences relative to the doses measured at a motion amplitude of 0 cm (no motion).

**FIGURE 5:**
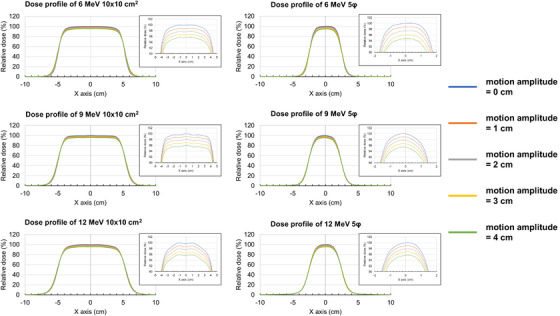
Comparison of dose profiles for each energy, field size, and amplitude (0, 1, 2, 3, and 4 cm) of the QUASAR heavy duty respiratory motion platform. The dose is expressed relative to that measured at isocenter with a motion amplitude of 0 cm.

Larger motion amplitudes of the QUASAR platform resulted in a larger field size and penumbra at the *d*_max_ (Table 4 and Figure 5). For electron beams of 6, 9, and 12 MeV with a 5 cm circular aperture, motion amplitudes of 0–3 cm resulted in expansions of the field by 0.5, 0.7, and 0.7 mm, respectively; for a 10 × 10 cm^2^ field, the expansions were 1.7 mm, 1.6 mm, and 1.6 mm, respectively. Additionally, for 6, 9, and 12 MeV with a 5 cm circular aperture, motion amplitudes of 0–3 cm resulted in penumbra expansions of 2.1, 1.5, and 1.0 mm, respectively; for a 10 × 10 cm^2^ field, the expansions were 1.6, 1.4, and 1.1 mm, respectively.

**TABLE 4:** Comparison of the differences in the irradiation field and penumbra sizes at the maximum depth (*d*_max_) for each energy, field size, and amplitude of the QUASAR platform motion, relative to a motion amplitude of 0 cm (no motion).

		The differences of the irradiation field size on X‐axis (mm)				The differences of the penumbra on X‐axis (mm)			
Energy	Field size (cm^2^)	Motion amplitude 1 cm	Motion amplitude 2 cm	Motion amplitude 3 cm	Motion amplitude 4 cm	Motion amplitude 1 cm	Motion amplitude 2 cm	Motion amplitude 3 cm	Motion amplitude 4 cm
6 MeV	5 cm circular aperture	0.1	0.3	0.5	0.7	0.6	1.3	2.1	2.6
9 MeV		0.2	0.5	0.7	0.9	0.5	0.9	1.5	2.0
12 MeV		0.2	0.5	0.7	0.9	0.3	0.6	1.0	1.2
6 MeV	10 × 10	0.5	1.1	1.7	2.1	0.5	1.0	1.6	2.1
9 MeV		0.5	1.1	1.6	2.1	0.4	0.9	1.4	1.9
12 MeV		0.6	1.1	1.6	2.1	0.3	0.7	1.1	1.5

## 4 DISCUSSION

This study is the first to systematically quantify the effects of extended SSDs and respiratory motion‐induced SSD variations on electron beam therapy. The results provide crucial insights for improving the precision of electron beam therapy, particularly in the treatment of keloids and superficial tumors, and have immediate clinical applicability. Consequently, for patients undergoing electron beam therapy targeting superficial thoracic and abdominal lesions, if respiratory‐induced SSD variations surpass 3 cm, the implementation of mitigation strategies becomes imperative. Such strategies may involve (1) abdominal compression to reduce respiratory motion, (2) breath‐hold techniques, or (3) gated irradiation. This research provides quantitative results to support clinical decisions regarding the necessity of these interventions.

First, the study assessed the impact of an extended SSD on treatment accuracy. It was confirmed that increasing the SSD beyond 100 cm can markedly reduce the dose at the *d*_max_. Notably, with a 6 MeV electron beam and a small field size (5 cm circular aperture), extending the SSD to 105 cm resulted in a dose reduction of 13.42%. This indicates that the impact of SSD extension is most pronounced for low‐energy beams and small field sizes, conditions under which the electron beam does not follow the inverse‐square law. Specifically, the lower the energy of the electron beam and the smaller the field size, the larger the scattering angle of the radiation.^3^, ^5^, ^7^, ^9^, ^10^ Furthermore, for low‐energy electron beams, the intensity of the radiation decreases sharply as a function of the distance traveled.^3^, ^5^, ^9^, ^10^, ^14^ Currently, such SSD variations are not accounted for in most treatment planning systems (TPSs); hence, clinicians must develop in‐house protocols or use empirical corrections.

Second, this study showed that extending the SSD also enlarges the field and penumbra at the *d*_max_. For example, for a 6 MeV electron beam with a 5 cm circular aperture, extending the SSD from 100 to 103 cm increased the field size by 1.2 mm and the penumbra by 2.6 mm. Because a lower energy electron beam undergoes more scattering within a material, an extended SSD results in the energy being distributed over a wider area, thereby expanding the penumbra.^3^, ^14^, ^15^ To ensure treatment accuracy, these findings suggest that treatment planning must consider the expansion of the field size and penumbra when the SSD is changing during a treatment.

We also evaluated the impact of respiratory motion‐induced SSD variations on treatment accuracy. For a respiratory motion amplitude of 3 cm, the dose at the *d*_max_ deviated by > 3% from that measured in the absence of motion for all energies and field sizes. For a respiratory motion amplitude of 4 cm, the largest dose difference of 5.36% was observed for a 6 MeV electron beam with a 5 cm circular aperture. Furthermore, a larger amplitude of the QUASAR platform motion resulted in larger field and penumbra sizes at the *d*_max_. For example, for a 6 MeV electron beam with a 5 cm circular aperture, a motion amplitude of 3 cm expanded the field by 0.5 mm and the penumbra by 2.1 mm compared with their respective sizes in the absence of motion. Although we focused mainly on SSDs > 100 cm, shortening the SSD to < 100 cm is expected to increase the dose at the *d*_max_ and decrease the field and penumbra. While combining data from Table 1 to Table 3 might suggest a theoretical possibility of offsetting respiratory‐induced dose variations by adjusting the setup SSD (e.g., targeting the midpoint of the respiratory cycle), we consider this approach clinically challenging and associated with high uncertainty for several reasons. Firstly, altering the SSD from the standard 100 cm, particularly shortening it, could reduce the field size and penumbra, potentially compromising target coverage. Secondly, as detailed in our Limitations section, the irregularity of actual patient respiration and the difficulty in accurately identifying the respiratory midpoint would make reproducible daily setups extremely challenging. Such inconsistencies could potentially introduce unforeseen dosimetric errors.

These results highlight the necessity for real‐time respiratory motion monitoring and dose correction technologies that account for respiration‐induced dose variations. Established strategies, such as abdominal compression, have limitations in effectively immobilizing the moving irradiated site. As a promising direction for future research, mitigating dose variations from respiratory motion‐induced SSD changes may be effectively addressed with four‐dimensional computed tomography (4D‐CT) like breath‐hold or gated irradiation. Meanwhile, minimizing variations in penumbra and field size could also be achieved by forming the irradiation field directly on the patient's skin. Furthermore, it is noteworthy that in many facilities, the SSD is set using CT images acquired during free breathing. Consequently, the treatment SSD can vary from the planned SSD due to respiratory motion, potentially offsetting output differences. This effect warrants further investigation using 4D‐CT. Building on the concept of skin‐level collimation, a technology like real‐time variable‐shape tungsten rubber (STR) is capable of forming an irradiation field on the skin surface and tracking movement.^16^, ^17^ Therefore, it might minimize the penumbral and field size variations caused by an extended SSD. However, this approach remains speculative and requires further investigation.

The limitations of this study should also be acknowledged. Firstly, it assumed a consistent respiratory motion pattern in healthy subjects. This idealized model does not account for the significant inter‐ and intra‐patient variability in respiratory patterns (e.g., in depth, rate, and rhythm) or unpredictable intra‐fraction movements such as coughing, which could lead to more complex dose distribution changes not fully assessed here. Secondly, the study utilized a maximum dose rate of 1000 MU/min for the electron beam. Lower dose rates, prolonging irradiation time, could intensify the impact of irregular respiratory motion, potentially magnifying observed effects such as dose reduction at the *d*_max_ and enlargement of the field and penumbra. Additionally, while efforts were made to accurately set up the phantom, the inherent uncertainties in daily clinical setup reproducibility, especially with extended SSDs, could further influence dosimetric accuracy and warrant future investigation. Future evaluations must therefore consider patient‐specific breathing patterns and these other variabilities. Additionally, it is important to acquire 4D‐CT images and subsequently investigate, using the TPSs, the impact of respiration‐induced SSD variations on dose calculations. Such an approach, based on the results of this study, holds promise for improving electron beam therapy. Our findings will significantly contribute to improving the precision of electron beam therapy. For instance, incorporating real‐time respiratory motion monitoring and instant dose correction technologies could substantially enhance treatment efficacy. Moreover, the insights gained from this study can be applied to other superficial tumor treatments affected by respiratory motion and extended SSDs in electron beam therapy.

## 5 CONCLUSIONS

To our knowledge, this study is the first to elucidate the detailed effects of extended SSDs and respiratory motion on electron beam therapy. By providing new insights for enhancing treatment precision, the study may support improvements in electron beam therapy in clinical practice. Respiratory motion management may be warranted when applying electron beam therapy to sites experiencing respiratory motion‐induced SSD variations > 3 cm.

## AUTHOR CONTRIBUTIONS

T. Ito, H. Kosaka, Y. Yanagi, Y. Sakai, and H. Monzen were associated with concept and design. T. Ito, H. Kosaka, Y. Yanagi, and Y. Sakai took the measurements. T. Ito, Y. Yanagi, and Y. Sakai analyzed the data. T. Ito, H. Kosaka, and H. Monzen prepared the manuscript. All authors read and approved the final manuscript.

## CONFLICT OF INTEREST STATEMENT

The authors have nothing to disclose.

## Data Availability

Authors will share data upon request to the corresponding author.

## References

[1] Liu EK , Cohen RF , Chiu ES . Radiation therapy modalities for keloid management: a critical review. J Plast Reconstr Aesthetic Surg. 2022;75(8):2455–2465. doi:10.1016/j.bjps.2022.04.099 35817711

[2] Maarouf M , Schleicher U , Schmachtenberg A , Ammon J . Radiotherapy in the management of keloids: clinical experience with electron beam irradiation and comparison with X‐ray therapy. Strahlenther Onkol. 2002;178(6):330–335. doi:10.1007/s00066-002-0935-6 12122789

[3] Cygler J , Li XA , Ding GX , Lawrence E . Practical approach to electron beam dosimetry at extended SSD. Phys Med Biol. 1997;42(8):1505–1514. doi:10.1088/0031-9155/42/8/003 9279902

[4] Saw CB , Pawlicki T , Korb LJ , Wu A . Effects of extended SSD on electron‐beam depth‐dose curves. Med Dosim. 1994;19(2):77–81. doi:10.1016/0958-3947(94)90075-2 7916979

[5] O'Shea TP , Foley MJ , Rajasekar D , et al. Electron beam therapy at extended source‐to‐surface distance: a Monte–Carlo investigation. J Appl Clin Med Phys. 2008;9(4):57–67. doi:10.1120/jacmp.v9i4.2811 PMC5722354 19020487

[6] Das IJ , McGee KP , Cheng C‐W . Electron beam characteristics at extended treatment distances. Med Phys. 1995;22(10):1667–1674. doi:10.1118/1.597431 8551993

[7] Bayatiani MR , Fallahi F , Aliasgharzadeh A , Ghorbani M , Khajetash B , Seif F . Determination of effective source to surface distance and cutout factor in small fields in electron beam radiotherapy: a comparison of different dosimeters. Polish J Med Phys Eng. 2020;26(4):235–242. doi:10.2478/pjmpe-2020-0028

[8] Khan FM , Doppke KP , Hogstrom KR , et al. Clinical electron‐beam dosimetry: report of AAPM radiation therapy committee task group no.25. Med Phys. 1991;18(1):73–109. 1901132 10.1118/1.596695

[9] Rajasekar D , Datta NR , Maria Das KJ , Ayyagari S . Electron beam therapy at extended SSDs: an analysis of output correction factors for a Mitsubishi linear accelerator. Phys Med Biol. 2002;47(18):3301–3311. doi:10.1088/0031-9155/47/18/303 12375822

[10] Khan FM , Sewchand W , Levitt SH . Effect of air space on depth dose in electron beam therapy. Radiology. 1978;126(1):249–251. doi:10.1148/126.1.249 619419

[11] Yanagi Y , Monzen H , Kubo K , et al. Comparison of the characteristics of two types of parallel‐plate ionization chamber under small‐field electron irradiation. Anticancer Res. 2023;43(5):1967–1972. doi:10.21873/anticanres.16356 37097686

[12] Thwaites DI , DuSautoy AR , Jordan T , et al. The IPEM code of practice for electron dosimetry for radiotherapy beams of initial energy from 4 to 25 MeV based on an absorbed dose to water calibration. Phys Med Biol. 2003;48(18):2929–2970. doi:10.1088/0031-9155/48/18/301 14529204

[13] Royal College of Physicians . National Early Warning Score (NEWS): Standardizing the Assessment of Acute‐Illness Severity in the NHS. Report of a Working Party. Royal College of Physicians, London; 2012.

[14] Ibbott GS . Radiation dosimetry: electron beams with energies between 1 and 50 MeV (ICRU Report No. 35). Med Phys. 1985;12(6):813–813. doi:10.1118/1.595780

[15] Li XA , Rogers DWO . Electron mass scattering powers: Monte–Carlo and analytical calculations. Med Phys. 1995;22(5):531–541. doi:10.1118/1.597582 7643788

[16] Kawai Y , Tamura M , Amano M , Kosugi T , Monzen H . First clinical experience of tungsten rubber electron adaptive therapy with real‐time variable‐shape tungsten rubber. Anticancer Res. 2021;41(2):919–925. doi:10.21873/ANTICANRES.14845 33517298

[17] Monzen H , Tamura M , Kijima K , et al. Estimation of radiation shielding ability in electron therapy and brachytherapy with real time variable shape tungsten rubber. Phys Medica. 2019;66:29–35. doi:10.1016/j.ejmp.2019.09.233 31550531

